# The role, mechanism, and application of RNA methyltransferase METTL14 in gastrointestinal cancer

**DOI:** 10.1186/s12943-022-01634-5

**Published:** 2022-08-16

**Authors:** Bin Shi, Wei-Wei Liu, Ke Yang, Guan-Min Jiang, Hao Wang

**Affiliations:** 1 Department of Anorectal Surgery, The First Affiliated Hospital of USTC, Division of Life Sciences and Medicine, University of Science and Technology of China, Heifei, China; 2 School of Basic Medical Sciences, Shandong University, Jinan, China; 3 School of Clinical Medicine, Clinical College of Anhui Medical University, Hefei, China; 4 Department of Clinical Laboratory, The Fifth Affiliated Hospital, Sun Yat-Sen University, Zhuhai, China; 5 Department of Clinical Laboratory, The First Affiliated Hospital of USTC, Division of Life Sciences and Medicine, University of Science and Technology of China, Hefei, China; 6 Core Unit of National Clinical Research Center for Laboratory Medicine, Heifei, China

**Keywords:** Gastrointestinal cancer, RNA modification, m6A, METTL14

## Abstract

Gastrointestinal cancer is the most common human malignancy characterized by high lethality and poor prognosis. Emerging evidences indicate that N6-methyladenosine (m6A), the most abundant post-transcriptional modification in eukaryotes, exerts important roles in regulating mRNA metabolism including stability, decay, splicing, transport, and translation. As the key component of the m6A methyltransferase complex, methyltransferase-like 14 (METTL14) catalyzes m6A methylation on mRNA or non-coding RNA to regulate gene expression and cell phenotypes. Dysregulation of METTL14 was deemed to be involved in various aspects of gastrointestinal cancer, such as tumorigenesis, progression, chemoresistance, and metastasis. Plenty of findings have opened up new avenues for exploring the therapeutic potential of gastrointestinal cancer targeting METTL14. In this review, we systematically summarize the recent advances regarding the biological functions of METTL14 in gastrointestinal cancer, discuss its potential clinical applications and propose the research forecast.

## Introduction

Increasing studies demonstrated that epigenetics plays a crucial role in cancer occurrence and progression [1, 2]. Different to genetic alterations, epigenetic modifications are reversible and inheritable processes which regulate gene expression without DNA sequences changes [3, 4]. Although the scope of epigenetics is not fully explored, it is commonly defined as chemical modifications, including chromatin rearrangement, DNA and RNA methylation, non-coding RNA and histone modification [5]. Previous reports mainly focused on the biological functions of DNA methylation, non-coding RNAs regulation and histone modification [6–8]. Recently, mounting studies have identified more than 100 kinds of chemical modifications in RNA, which exploits a new research field of epigenetic regulation controlled by RNA modification [9–11]. RNA methylation is the main form of RNA modifications, including N6-methyladenosine (m6A), m1A, 1-methylguanosine (m1G), m2G, m6G, m7G, 5-methylcytosine (m5C), 2ʹ-O-methylation (Nm), pseudouridine (Ψ) and Inosine (I), among which m6A modification is the most abundant kind accounting for approximately half of all RNA methylation modifications [5, 12–14]. m6A modification exists in nearly all eukaryotes and in a part of viruses, yeasts, bacteria, and plants [12]. m6A binding sites are found in the RRACH sequence (*R* = A/G, H = A/C/U) and are mainly enriched in the 3’ untranslated regions (UTRs) near the stop codon of mRNA exon [12, 15]. Remarkably, m6A mediated-RNA epigenetics modification plays an important role in controlling physiological activities, such as embryonic stem cell differentiation, DNA repair, meiosis, tissue remodeling, and circadian rhythm, etc. [11, 16]. Dysregulation of m6A modification gives rise to multiple pathological processes, including tumorigenesis and development [14, 17, 18].

### Composition of m6A

**Fig. 1:**
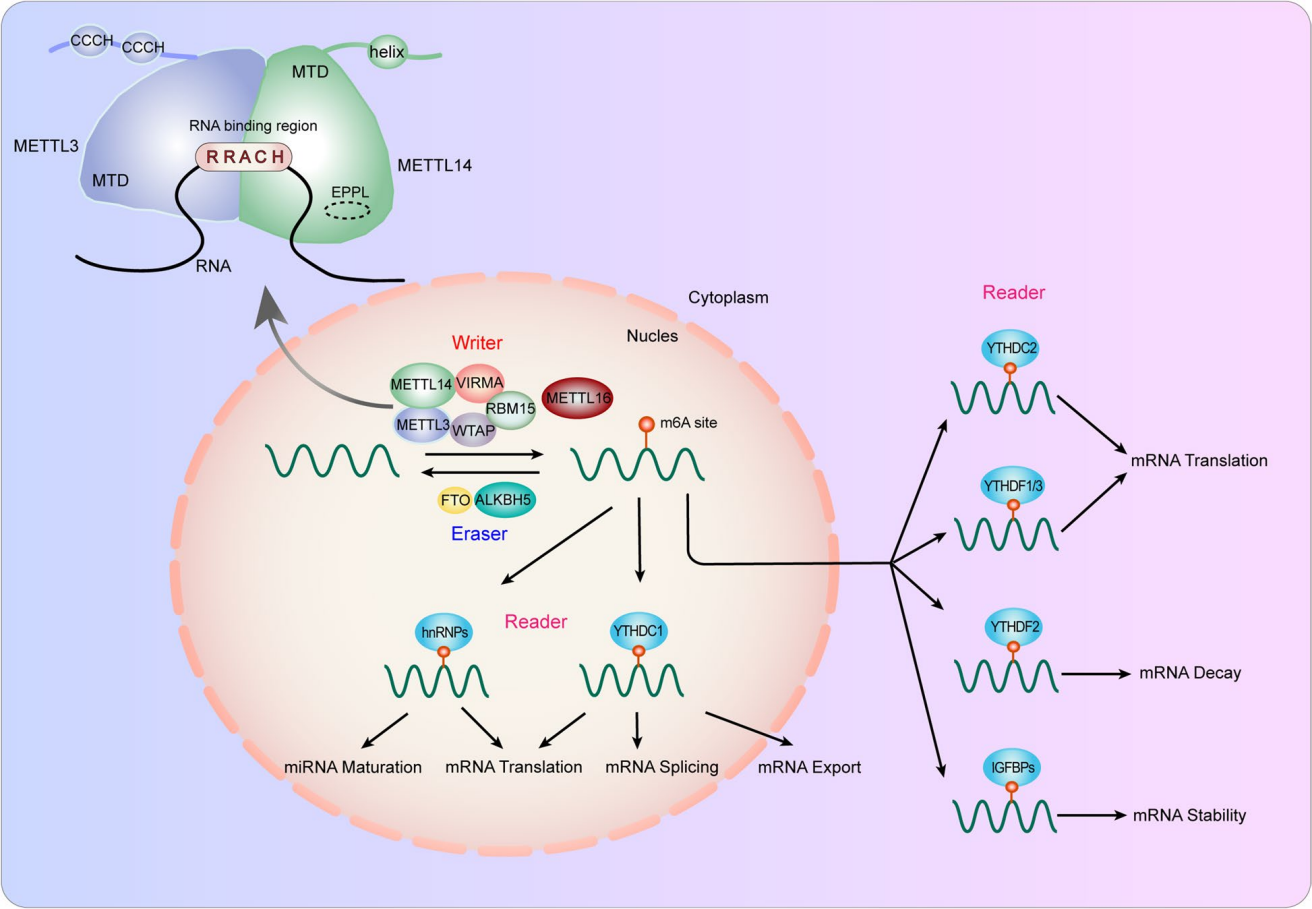
The composition and function of m6A modification. The m6A modification is installed by writers, including METTL3, METTL14, WTAP, RBM15, VIRMA and METTL16. FTO and ALKBH5 are m6A erasers that remove m6A modifications. Readers are required to recognize m6A and exert post-transcriptional regulation. Writers, erasers, and readers synergistically regulate RNA splicing, export, translation, decay, and stability

As a dynamic and reversible process, m6A modification can be catalyzed by m6A methyltransferases (“writer”) and eliminated by demethylases (“eraser”) [19–21]. Moreover, RNA-binding proteins (“reader”) specifically recognize and bind to m6A sites to regulate fate of RNAs (Fig. 1) [16, 19, 22].

### Writer

“Writer” regulators traditionally consist of methyltransferase-like 3/14/16 (METTL3/14/16), WT1-associated protein (WTAP), zinc finger CCCH-type containing 13 (ZC3H13), virlike m6A methyltransferase-associated (VIRMA/KIAA1429), RNA-binding motif protein 15/15B (RBM15/15B), and Fl(2)d-associated complex component (Flacc) [15, 22–29]. Among them, METTL3 is the first identified m6A methyltransferase and exerts catalytic function with the assistance of METTL14, which stabilizes METTL3 and recognizes target RNAs [30–33]. METTL3 and METTL14 form a stable methyltransferase complex, while WTAP interacts with the heterodimer complex and ensures it to be localized in the nuclear spots and triggers catalytic activity [34–36]. METTL16 can function alone and control m6A modification in mRNAs, U6-snRNA and long noncoding RNAs [27, 29, 37–39]. RBM15, VIRMA and ZC3H13 modulate region-selective m6A methylation modification by binding to methyltransferase complex and localizing it to special RNA sites [40–42].

### Eraser

m6A methylation can be eliminated via demethylation, which is mediated by demethylases, also called “eraser”, including Fat mass and obesity-associated protein (FTO) and AlkB homolog 3/5 RNA demethylase (ALKBH3/5) [17, 43]. FTO is identified as the first “eraser” and mainly influence mRNA stability, translation and splicing by regulating m6A demethylation [44, 45]. As the homologue of FTO, ALKBH3 and ALKBH5 principally mediate the transport, metabolism, and assembly of mRNA [46–48]. These erasers promote the transformation of m6A into N6-hydroxymethyladeosine and N6-formyladenosine successively, which is finally hydrolyzed into adenosine [12, 17].

### Reader

In addition, another essential group of regulators of m6A modification is “reader”, which can recognize and bind to m6A methylated targets to induce various biological phenotypes. The “reader” mainly consists of YTH domain family of proteins (YTHDC1/2, YTHDF1/2/3) [49–55], IGF2 mRNA binding protein (IGF2BP1/2/3) [56–59], the heterokaryotic nuclear RNA protein family (HNRNPC, HNRNPG) [60–62], and eukaryotic initiation factor 3 (eIF3) [63, 64], which affect m6A methylation by modulating RNA metabolism [16].

### Function of m6A

"Writers", "erasers" and “readers” work together to effectively catalyze, remove and recognize m6A methylation and establish a reversible and dynamic balance of m6A modification. mRNA, miRNAs, and long noncoding RNAs can all be regulated by m6A methylation, which controls RNA stability, decay, translation, splicing, transport, localization, and RNA–protein interactions (Fig. 1) [20, 65–67].

### Splicing

m6A modification can modulate pre-mRNA splicing by interacting with different splicing factors. FTO preferentially binds adjacent to the alternative splicing exon and polyA sites, thus depresses recruitment of serine/arginine-rich splicing factor 2 (SRSF2) and induces exon 6 skipping [44, 68]. ALKBH5 can promote the phosphorylation of ASF/SF2, and the hyper-phosphorylated ASF/SF2 participates in splicing [46]. It has been reported that downregulation of m6A writers interfered splicing and gene expression [69, 70]. Also, loss of hnRNPC/hnRNPG can change the splicing pattern in an m6A-dependent way [62].

### Nuclear export

Previous studies confirmed that ALKBH5 could restrain nuclear export, determining the subcellular location of mRNAs. Mechanistically, ALKBH5 can reduced the hypo-phosphorylated form of ASF/SF2, which promotes mRNA export mediated by TAP-p15 complex [71]. YTHDC1 facilitates nuclear export by promoting the binding of RNA to nuclear RNA export factor 1 (NXF1) and export adaptor protein SRSF3 [72]. Fragile X mental retardation protein (FMRP), another reader, was identified to be indispensable in CRM1-mediated nuclear export [73].

### Translation

METTL3 can regulate translation via different readers specifically recognizing m6A sites. It can also exert regulatory role independent on methyltransferase activity, by interacting with eIF3h to promote translation [74, 75]. Recently, METTL16 has also been confirmed to regulate translation in both methyltransferase activity-dependent and -independent manner [27]. Notably, YTHDF proteins play important roles in modulating translation. YTHDF1 can promote the cap-dependent translation initiation by participating in the formation of loop structure with eIF4G and eIF3 and recruitment of ribosomes [49]. Besides, YTHDF1 can facilitate the expression of eIF3C in an m6A-dependent way [76]. In synergism with YTHDF1, YTHDF3 promotes translation via interacting with 40S and 60S ribosome subunits [77]. YTHDF3 also improves the translation efficiency of ITGA6 and promotes malignant progression of bladder cancer [78]. YTHDC2 can boost translation with its helicase activity, independent on m6A modification, which is enhanced by 5' → 3' exoribonuclease XRN1 [79].

### Stability

m6A modification serves as a double-edged sword in regulating mRNA stability. YTHDF2 plays a vital role in RNA degradation by recruiting the deadenylase complex CCR4-NOT [80]. And YTHDF3 cooperates with YTHDF2 to facilitate mRNA degradation [81]. For instance, YTHDF2 recognizes the methylation of suppressor of cytokine signaling 2 (SOCS2) and arrestin domain-containing protein 4 (ARRDC4) and induces their mRNA degradation thus enhances metastasis and dissemination of cancer cells [82, 83]. YTHDF1 can induce the degradation of MAT2A mRNA by binding to m6A sites in the 3’-UTR [84]. Another group of readers, IGF2BP1/2/3 can enhance mRNAs stability via KH domain binding to target m6A sites [56]. Moreover, FTO is reported to increase the stability of MYC mRNA by depressing the YTHDF2-mediated decay [85].

### METTL14

As a key allosteric activator of METTL3, METTL14 functions as the major m6A methyltransferase to regulate m6A modification on mRNA and non-coding RNA. Advances have been achieved in exploring the crucial roles and molecular mechanisms of METTL14 in multiple types of cancer, especially in gastrointestinal cancer, including liver cancer, colorectal cancer, gastric cancer, and pancreatic cancer. In this review, we will summarize the biological functions and underlying mechanisms of METTL14 in gastrointestinal cancer determined by the latest research progresses of our and other research teams, discuss the potential clinical applications and propose future research directions of METTL14 in gastrointestinal cancer.

### The structural basis of METTL14

METTL3 and METTL14 form a stable heterodimer in 1:1 ratio, the N-terminal extension of METTL14 interacts with METTL3 via loops and helixes (Fig. 1) [32, 33]. METTL14 has the homologous methyltransferases domain (MTD) as METTL3, but it owns a closed conformation of catalytic chamber without SAM binding sites. And METTL14 lacks the two CYS-CYS-HIS (CCCH)-type zinc binding motifs of METTL3, which also deprives its catalytic activity [33]. However, it exerts an essential structural role to support METTL3's catalytic function and METTL3 alone merely exhibits weak activity. Significantly, METTL14 provides an RNA-binding scaffold that plays a crucial role in recognizing and binding substrate RNAs [86]. The RGG repeats of the METTL14 C-terminus are supposed to contribute to the recognition of RNAs [87]. Similar to METTL3, METTL14 preferentially recognizes RNA with the “RRACH” (*R* = A/G, H = A/U/C) sequences [30], but the priority mechanism remains unclear.

### The function role of METTL14 in gastrointestinal cancer

**Fig. 2:**
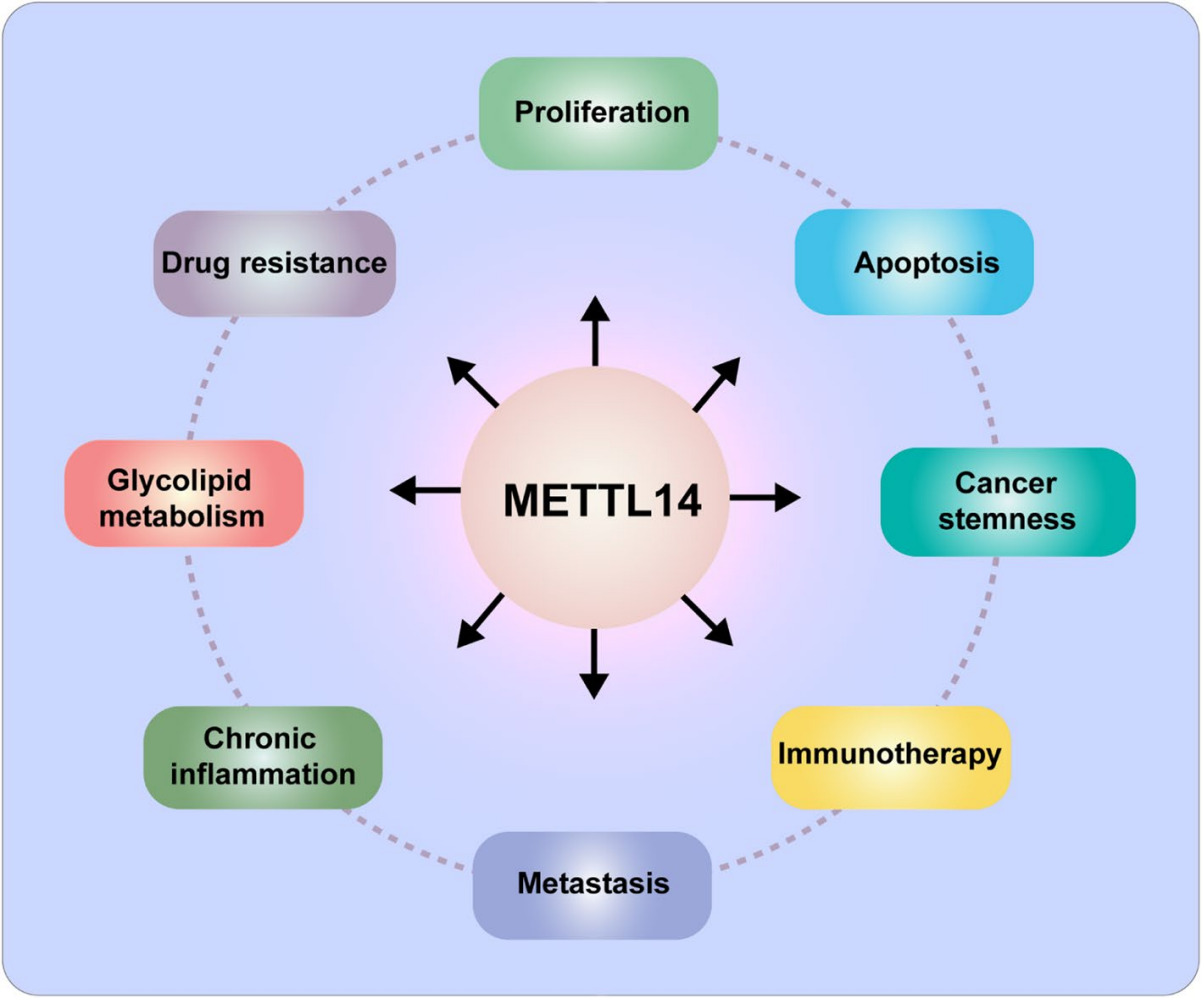
The biological function of METTL14 in cancer. METTL14 is involved in various processes of tumor development, including proliferation, metastasis, apoptosis, drug resistance, cancer stem cell like characteristic, immunotherapy, chronic inflammation, and glycolipid metabolism

Recent researches have demonstrated that dysregulation of METTL14 is tightly relative to the phenotypes involved in the malignant development of various cancer, including proliferation [88–90], metastasis [91–96], apoptosis [97–101], drug resistance [102–105], cancer stem cell like characteristic [106, 107], immunotherapy [21, 108, 109], chronic inflammation [110] and glycolipid metabolism (Fig. 2) [95]. Herein, we systematically summarize the recent advances of METTL14 in gastrointestinal cancer (Table 1).

**Table 1:** Role of METTL14 in gastrointestinal cancer

Cancer type	Role	Target	Upstream	Reader	Functions
HCC	Tumor suppressor	EGFR PI3K/AKT			Suppresses migration and invasion [111]
	Tumor suppressor	miR-126			Suppresses metastasis [92]
	Tumor suppressor	SLC7A11	HIF-1α	YTHDF2	Suppresses hypoxia blocked-ferroptosis [112]
	Tumor suppressor	USP48 SIRT6			Attenuate glycolysis and malignancy [113]
	Tumor suppressor	HNF3γ			Suppresses proliferation and sorafenib resistance [103]
	Oncogene	ACLY SCD1			Promotes FA synthesis and lipid accumulation [114]
CRC	Tumor suppressor	miR-375 YAP1/SP1			Suppresses migration, invasion, proliferation [94]
	Tumor suppressor	KLF4		IGF2BP2	Suppresses migration and invasion [115]
	Tumor suppressor	ARRDC4	HuR TCF4	YTHDF2	Suppresses metastasis [83]
	Tumor suppressor	SOX4	KDM5C H3K4me3	YTHDF2	Suppresses metastasis [116]
	Tumor suppressor	LincR XIST		YTHDF2	Suppresses proliferation and metastasis [93]
	Tumor suppressor	miR-149-3p			Suppresses inflammation and malignancy [110]
	Tumor suppressor	IFN-c/ STAT1/IRF1		YTHDF2	Promotes immune responses to anti-PD-1 therapy [108]
	Tumor suppressor	EBI3			Promotes antitumor response and CD8 + T cell infiltration [109]
	Tumor suppressor	ANKLE1		YTHDF1	Suppresses proliferation, and colony formation [117]
	Oncogene	PHLDB2			Promotes Cetuximab Resistance [105]
GC	Tumor suppressor	circORC5			Suppresses proliferation and invasion [118]
	Tumor suppressor	Wnt/ PI3K‐Akt			Suppresses proliferation and invasion [119]
	Tumor suppressor	PI3K/AKT/ mTOR			Suppresses proliferation and invasion [120]
	Oncogene	LINC01320			Promotes migration, invasion, proliferation [121]
PC	Oncogene	PERP			Suppresses growth and metastasis [91]
	Oncogene	CDA	P65		Promotes gemcitabine resistance [122]
	Oncogene	AMPKα/ ERK/mTOR			Suppresses apoptosis and autophagy [101]
	Tumor suppressor	PIK3CB		YTHDF2	Suppresses proliferation and invasion [123]
	Tumor suppressor		CLK1 SRSF5		Suppresses proliferation and metastasis [96]

### Liver cancer

**Fig. 3:**
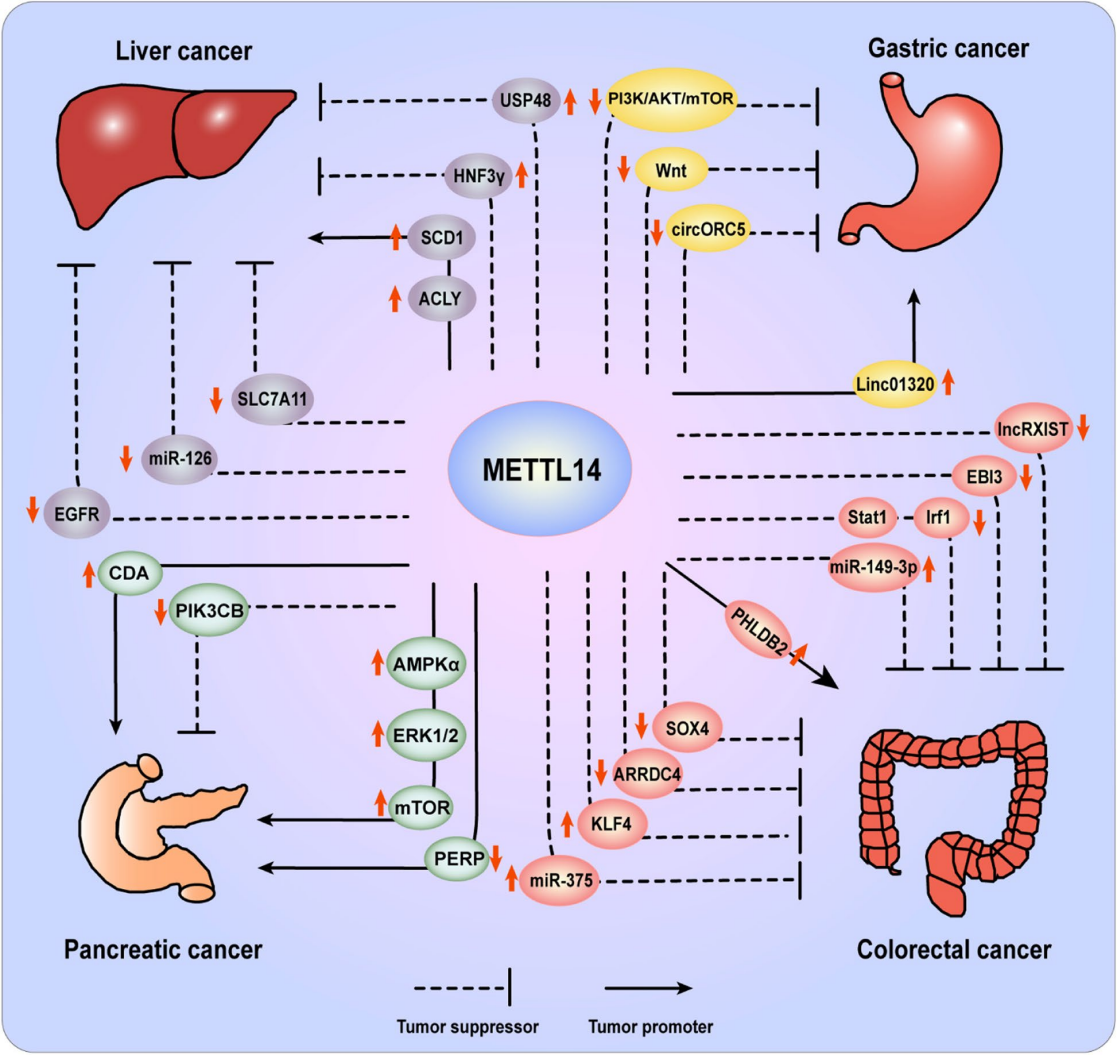
The targets and functions of METTL14 in gastrointestinal cancer. In liver cancer, METTL14 suppresses cancer development via downregulating EGFR, miR-126, SLC7A11 and upregulating USP48, HNF3γ, while promotes tumor progression via increasing SCD1 and ACLY expression. In GC, METTL14 suppresses cancer development via inhibiting circORC5, Wnt and PI3K/AKT/mTOR, while promotes tumor progression via upregulating Linc01320. In CRC, METTL14 suppresses cancer development via downregulating ARRDC4, SOX4, EBI3, STAT1/IRF1, LncRXIST and upregulating KLF4, miR-375, miR-149-3p, while promotes tumor progression via increasing PHLDB2 expression. In PC, METTL14 suppresses cancer development via downregulating PIK3CB, while promotes tumor progression via increasing CDA, AMPKα/ERK/mTOR, and downregulating PERP

Liver cancer (LC) is a common malignancy with the fourth lethality in cancers worldwide. The predominant form of LC is hepatocellular carcinoma (HCC), which accounts for ~ 80% of primary LC and present an increasing incidence globally [124]. Emerging reports have confirmed the significance of m6A modification in LC, and continuous efforts have been put to investigate the complicated molecular mechanism of abnormal m6A modification and dysregulation of m6A regulators in HCC. First of all, the expression level of METTL14 was identified to be obviously decreased in HCC, which closely correlated with clinicopathological factors, including tumor stage and prognosis (Fig. 3) [125–128]. Based on the analysis of data from The Cancer Genome Atlas (TCGA) and Gene Expression Omnibus (GEO), Liu et al. showed the opposite expression level and prognostic value of METTL14 and METTL3 in HCC [127]. Similarly, METTL14 was predicted to participate in HCC malignant progression by modulating the m6A-modified transcripts, such as cysteine sulfonate decarboxylase (CSAD), glutamic oxalacetic transaminase (GOT2), and SOCS2 [126]. Through overlapping RNA-sequencing and m6A-sequencing data, epidermal growth factor receptor (EGFR) was identified as the direct target of METTL14. Knockdown of METTL14 activates EGFR/PI3K/AKT signaling and thus promotes epithelial-mesenchymal transition (EMT), migration and invasion of HCC cells [111]. In metastatic HCC, METTL14 interacts with the microprocessor protein DiGeorge syndrome critical region 8 (DGCR8) to suppress tumor metastasis. Mechanistically, METTL14 enhances the engagement of pri-miR126 by DGCR8 and promotes the subsequent processing into miRNA126, which was recognized as a metastasis suppressor. What’s more, the researchers verified the suppressive role of miR126 in HCC cell metastasis using miR126 mimic and inhibitor [92]. Beyond inhibiting metastasis, METTL14 serves as a tumor suppressor involved in various biological processes. METTL14 facilitates hypoxia-blocked ferroptosis of HCC cells by catalyzing m6A modification at the mRNA 5’UTR of solute carrier family 7 member 11 (SLC7A11), then promotes YTHDF2-dependent degradation of SCL7A11 transcripts [112]. METTL14-triggered m6A methylation also inhibits the degradation of ubiquitin specific peptidase 48 (USP48) mRNA, which can deubiquitylate and stabilize sirtuin 6 (SIRT6) to suppresses glycolysis and HCC tumorigenesis. The METTL14-USP48-SIRT6 signaling may be a potential therapeutic strategy for HCC in the future [113]. In addition, reduced METTL14 level in HCCs decreases the stability of m6A-modified hepatocyte nuclear factor-3γ (HNF-3γ) mRNA, since decreased m6A level impairs IGF2BPs-mediated stabilization of mRNA. Reduced HNF3γ expression not only leads to HCC proliferation by inhibiting the differentiation of HCC cells and liver cancer stem cells, but also downregulates organic anion-transporting polypeptide 1B1 (OATP1B1) and 1B3 (OATP1B3) expression and thus impedes sorafenib uptake, resulting in the decreased sensitivity of HCC cells to sorafenib [103]. However, Yang et al. proposed the opposite role of METTL14 in HCC, they detected upregulated level of METTL14 in both HCC cells and patient samples. It was demonstrated that overexpressed METTL14 stabilized m6A-modified ATP citrate lyase (ACLY) and stearoyl-CoA desaturase 1 (SCD1) mRNA to increase their expression, thereafter aggravated FA synthesis and lipid accumulation, which contributed to DNA damage, chronic inflammation, cell apoptosis, excessive compensatory cell proliferation in livers, further developing non-alcohol Fatty Liver Disease (NAFLD) and HCC [114]. In conclusion, these findings suggested the important impact of METTL14 on LC.

### Colorectal cancer

Colorectal cancer (CRC) is a malignant tumor worldwide with an increasingly high incidence and mortality. Recurrence and metastasis are stubbornly major barriers to the treatment of CRC patients. According to the statistics, there are approximately 945,000 new cases and about 700,000 deaths of CRC every year [129–131]. Despite the great research advances in CRC over the years, the molecular mechanisms underlying tumorigenesis and development are still elusive. Recently, growing evidences revealed that METTL14-mediated m6A modification plays a vital role in controlling the progression of CRC (Fig. 3). Experimental studies and bioinformatics confirmed that METTL14 is highly expressed in CRC compared with normal tissues, and high expression level of METTL14 is closely associated with the better prognosis of CRC patients [83, 132, 133]. METTL14 can inhibit CRC metastasis and proliferation through multiple pathways and mechanisms. For example, Chen et al. discovered that the overexpressed METTL14 dramatically enhanced m6A level of CRC cells and suppressed CRC proliferation and metastasis in vitro, while METTL14 loss exerts opposite roles. Mechanistically, METTL14 modulates the processing of pre-miR-375 by DGCR8 and increases miR-375 level via a m6A dependent manner, which subsequently inhibits CRC growth and metastatic capability through downregulating Yes-associated protein 1 (YAP1) and SP1 respectively [94]. Wang et al. found that METTL14 can upregulate the expression of tumor suppressor protein Kruppel-like factor 4 (KLF4) to inhibit the invasion and metastasis of CRC cells. During this process, IGF2BP2 was involved in identifying m6A methylation sites of KLF4 to stabilize its mRNA [115]. In addition, our recent study demonstrated that METTL14 served as an independent predictor of CRC survival and suppressed CRC metastasis in vivo and in vitro. Through transcriptomic sequencing (RNA-seq) and methylated RNA immunoprecipitation sequencing (MeRIP-seq), METTL14 was identified to downregulate ARRDC4 by promoting its mRNA degradation depending on YTHDF2 recognition. Furthermore, the EMT related transcriptional factor ZEB1 was elevated by increased ARRDC4 in METTL14 deficient CRC cells, promoting metastasis of CRC [83]. Chen et al. also indicated METTL14 as a prognostic factor in CRC. They proved that METTL14 and YTHDF2 synergistically regulated m6A methylation modification and decreased the expression of SRY-box transcription factor 4 (SOX4), thereby abrogated EMT and PI3K/AKT signaling pathway, ultimately inhibited migration, invasion, and metastasis of CRC both in vivo and on vitro [116]. Another research confirmed that METTL14 blocked the metastasis and proliferation of CRC by decreasing oncogenic lncRNA XIST expression relying on YTHDF2-mediated degradation [93]. Recently, Tian et al. showed that the variant rs8100241[A] of tumor suppressor ankyrin repeat and LEM domain-containing protein 1 (Ankle1) could be more efficiently catalyzed by METTL14 and recognized by YTHDF1, thus upregulating m6A methylation level and protein expression of ANKLE1, which correlates with a reduced risk of CRC by suppressing tumor malignant proliferation and maintaining the genomic stability [117]. Moreover, besides metastasis and proliferation, METTL14 can modulate other malignant phenotypes. Dong et al. proved that METTL14 depletion in CRC-associated macrophages can induce epstein-Barr virus induced 3 (EBI3) upregulation in an m6A dependent manner mediated by YTHDF2, contributing to CD8^+^ T cells dysfunction, thereafter accelerating malignant progression of CRC, which was verified by mouse models and clinical samples [109]. Nevertheless, Wang et al. observed that loss of METTL14 elevated the response of CRC to programmed cell death-1 (PD-1) therapy. Reduced METTL14 can promote the amount of CD8 + T cells to secrete interferon-γ (IFN-γ), Chemokine (C-X-C motif) ligand 19 (CXCL19) and CXCL10, via enhancing the stability of Signal transducer and activator of transcription 1(STAT1) and interferon regulatory factor 1 (IRF1) mRNA dependent on YTHDF2, both of which are involved in IFN-γ signaling and anti-PD1 response. This enlightened that METTL14 could be a potential therapeutic target in mismatch-repair-proficient or microsatellite instability-low (pMMR-MSI-L) CRC [108]. Furthermore, Cao et al. revealed that Enterotoxigenic Bacteroides fragilis (ETBF) inhibited METTL14 to reduce m6A modified splicing of pri-miR-149, leading to downregulation of miR-149-3p. Subsequently, the decreased miR-149-3p not only induced PHF5A-mediated KAT2A RNA alternative splicing to promote tumorigenesis of CRC, but also contributed to the differentiation of Th17 cells resulting in intestinal inflammation [110]. However, Luo et al. showed the proto-oncogene role of METTL14 in CRC. They found that oxidative stress induced by chemotherapeutic drug could upregulate METTL14, which elevated the expression of Pleckstrin homology-like dom ain family B member 2 (PHLDB2). Increased PHLDB2 enhanced EGFR stability, contributing to chemo-resistance of CRC to cetuximab [105]. In summary, these studies proved the close connection between METTL14 and CRC progression, suggesting that METTL14 may be a potential therapeutic target for CRC treatment.

### Gastric cancer

Gastric cancer (GC) is a common malignant tumor worldwide. Although the incidence and mortality of GC display a downward trend in recent years, there are a mass of GC patients in China with the third mortality rate in cancer death, remarkably higher than other countries and regions [134–137]. The fact that epigenetics plays a significant role in GC progression has been widely confirmed. However, the clinicopathological functions and molecular mechanisms of m6A modification in GC remain largely unclear. To date, there has been no consensus on the role of METTL14 in GC (Fig. 3). Fan et al. showed that METTL14 was downregulated in GC tissues and associated with the poor survival in GC patients. METTL14 deficiency induced proliferation and metastasis of GC cells both in vivo and in vitro, while METTL14 overexpression harbored the opposite roles. Mechanistically, METTL14 triggered circORC5 m6A methylation modification to repress its expression, thus increased miR-30c-2-3p expression and whereafter downregulated AKT1 substrate 1 (AKT1S1) and eukaryotic translation initiation factor 4B (eIF4B), resulting in inhibition of GC tumorigenesis [118]. Liu et al. demonstrated that METTL14 was a tumor suppressor and potential biomarker of GC via bioinformatics analysis and clinical samples. METTL14 was downregulated in GC and exogenous expressed METTL14 repressed aggressive phenotype of GC by deactivating the PI3K/AKT/mTOR signaling axis [120]. In addition, Zhang et al. revealed that deficiency of METTL14 induced proliferation and invasion of GC cells by activating Wnt and PI3K‐Akt signal pathway in vitro. And they also found the potential correlation between m6A level and immunotherapy features and interferon signaling in METTL14-knockdown cells [119]. Nevertheless, Hu et al. expressed the opposite view that METTL14-mediated upregulation of long noncoding RNA Linc01320 can facilitate GC tumorigenesis in vitro. Linc01320 was found to downregulate miR-495-5p, leading to upregulated RAB19 in GC cells, which promotes GC cells proliferation, migration, and invasion in an unclear mechanism [121]. Conclusively, the biofunctions and regulation mechanisms of METTL14 in GC is rarely investigated and the research advances are limited. It is worth to extensively explore the value of METTL14 in GC in the future research.

### Pancreatic cancer

Pancreatic cancer (PC) is one of the most malignant tumors with a five-year survival rate of only 8% and largely PC patients die within seven years after surgery treatment [138, 139]. Since the mortality rates are on the rise, PC is predicted to become the second most common cause of cancer death by 2030 [140]. However, the underlying mechanisms of PC’s high lethality are still not well determined. Therefore, screening and identifying the critical molecules that regulate PC progression is meaningful for performing possible therapeutic strategies. Recently, emerging studies have reported the important role of m6A modification in PC (Fig. 3). Bioinformatics projections showed that the expression of METTL14 was closely related to overall survival of pancreatic ductal adenocarcinoma (PDAC) [141]. Based on TCGA database, Xu et al. established an independent risk prognostic signature of PC consisted of 5 m6A regulating genes, including METTL14, METTL3, KIAA1429, ALKBH5 and YTHDF1 [142]. Wang et al. determined that METTL14 served as an oncogene in PC. METTL14 was highly expressed in PC tissue and associated with the poor survival of PC patients. Increased METTL14 expression induced the degradation of p53 effector related to PMP-22 (PERP) via m6A dependent manner, contributing to the proliferation and metastasis of PC [91]. Interestingly, Chen et al. found that Cdc2-like kinases 1 (CLK1)/SR-like splicing factors5 (SRSF5) axis mediated aberrant exon skipping of METTL14, which leads to dysregulated m6A methylation modification and promotes the proliferation and metastasis of PDAC [96]. Moreover, Tian et al. showed that the variant rs142933486[G] allele of oncogene phosphatidylinositol-4,5-bisphosphate 3-kinase catalytic subunit beta (PIK3CB) was correlated with high level of m6A modification, which was catalyzed by METTL14 and recognized by YTHDF2. This could promote mRNA decay and decrease PIK3CB expression, leading to a reduced risk of PC [123]. A recent study showed that the expression of METTL14 was increased in gemcitabine resistant PC cells, while inhibition of METTL14 significantly enhanced the gemcitabine sensitivity of resistant PC cells by downregulating cytidine deaminase (CDA), an enzyme which can inactivate gemcitabine. And the downregulation of CDA is supposed to be mediated by regulation on the mRNA stability in an m6A-dependent manner [122]. Similarly, METTL14 knockdown promoted apoptosis and autophagy and enhanced sensibility of PC cells to cisplatin by repressing AMPKα, ERK1/2 and mTOR signal pathways, but the regulatory mechanisms of METTL14 and the exact roles of AMPKα and ERK1/2 in this process need further explorations [101]. In general, the role of METTL14 in PC is distinct from that in HCC, CRC, and GC. Since METTL14 acts as an oncogene in PC, it may be an effective therapeutic target for PC.

### Upstream regulators of METTL14

**Fig. 4:**
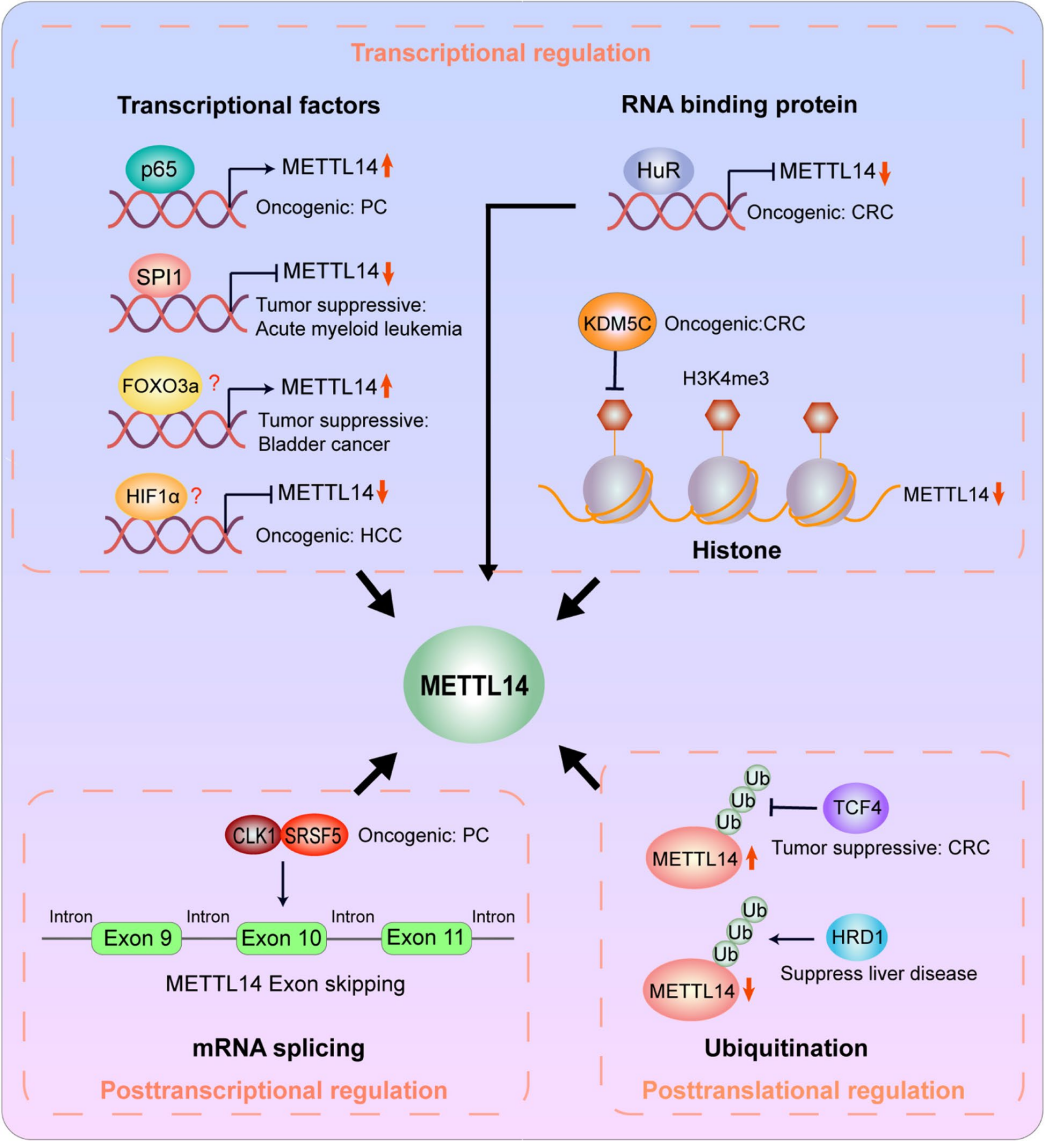
The upstream region of METTL14. KDM5C inhibits histone H3K4me3 and decrease METTL14 mRNA expression. The transcription factor SPI1 and HIF1α and RNA binding protein HuR inhibit METTL14 mRNA expression, while p65 and FOXO3a promote METTL14 mRNA expression. The SPI1, HuR and p65 can directly bind to the promoter of METTL14, however, the regulation mechanism of FOXO3a and HIF1α on METTL14 need to be further explored. In addition, CLK1 and SRSF5 can control METTL14 exon10 skipping. Moreover, TCF4 can inhibits ubiquitination of METTL14 and increases its expression. HRD1 is an E3 ubiquitin ligase of METTL14 and can promote ubiquitination of METTL14 and decreases its expression

Considering the important role of METTL14 in cancer progression, emerging studies have pay emphasis on the upstream regulatory mechanisms involved in the aberrant expression of METTL14 in cancer (Fig. 4).

### Transcriptional regulation

It was universally recognized that transcriptional factors and histone modification play the key role in regulating METTL14. In bladder cancer cells, knockdown of transcriptional factor forkhead box O3a (FOXO3a) conspicuously decreased METTL14 expression [143]. A recent study revealed that the transcriptional factor p65 was involved in upregulating METTL14 by targeting its promoter site in PC cells [122]. Moreover, Weng et.al showed that transcription factor PU.1 (Putative oncogene Spi-1, SPI1) functioned as a direct transcriptional suppressor of METTL14. SPI1 knockdown led to upregulation of METTL14 mRNA and protein in both normal and malignant hematopoietic cells, while SPI1 overexpression harbored the opposite effect in these cells [107]. In CRC, our latest findings proved that RNA-binding protein human antigen R (HuR) directly bound to METTL14 promoter and thus suppressed its expression [83]. In addition, Chen et al. indicated that (K)-specific demethylase 5C (KDM5C)-mediated demethylation of H3K4me3 could repress the transcription of METTL14 [116].

### Posttranscriptional regulation

Chen et al. found that CLK1/SRSF5 axis could regulate aberrant exon skipping of METTL14 [96]. Through transcriptome sequencing, METTL14 exon10 skipping regulated by the CLK1-SRSF5 axis was identified as the key alternative splicing event, promoting the m6A modification level and metastasis of PDAC cells.

### Posttranslational regulation

Recently, we demonstrated that transcriptional factor 4 (TCF4) depletion downregulated METTL14 expression via promoting its ubiquitination-mediated degradation in CRC. Furthermore, Wei et al. revealed that endoplasmic reticulum (ER) proteotoxic stress selectively promoted METTL14 expression through inhibiting its ubiquitination-mediated degradation by repressing HMG-CoA reductase degradation protein 1 (HRD1), an important E3 ubiquitin ligase of METTL14 [100].

Taken together, these findings illustrate that METTL14 abnormal expression can be affected by a series of transcription factors, histone modification and ubiquitination-mediated degradation. Investigation on the upstream regulatory mechanisms of METTL14 dysregulation helps us to better comprehend the biological role of METTL14 in cancers and provides possible therapeutic targets for anti-tumor therapy.

### Potential clinical application of METTL14

The above evidences emphasize that METTL14 is essential for tumorigenesis and development of gastrointestinal cancer, suggesting that METTL14 may be a promising biomarker for clinical diagnosis and potential therapeutic target of gastrointestinal cancer. The results from our and other laboratories showed that METTL14 was downregulated in CRC, HCC and GC, repressed tumor proliferation and metastasis and correlated negatively with tumor prognosis [83, 92, 93, 116, 118]. And we found that decreased METTL14 level was tightly associated with tumor stages of CRC. By the multivariate Cox regression analysis, METTL14 was identified as an independent prognostic factor for CRC patients [83]. Therefore. METTL14 may be a promising biomarker of aggressive CRC, HCC, and GC. However, it is obvious that more attention has been paid on exploring the functions and mechanisms of METTL14 in gastrointestinal cancer, while the expression of METTL14 in early stage of tumorigenesis need to be further investigated and confirmed. It is worthwhile to evaluate METTL14 as a biomarker for early diagnosis and prevention of gastrointestinal cancer in the future studies.

Immunotherapy has become one of the unprecedented therapeutic strategies for multiple malignant tumors by modulating the immune system of cancer patients. By targeting m6A modification, immune responses can be further significantly activated during antitumor immunotherapy. A recent study indicated that suppression of METTL14 mediated-m6A mRNA modification elevated the therapeutic effect of anti-PD-1 therapy in CRC. Inhibition of METTL14 not only promoted the proliferation and accumulation of cytotoxic tumor-infiltrating CD8 + T cells, but also induced the secretion of IFN-C, CXCL9, and CXCL10, thus enhanced immunotherapy efficacy and suppressed cancer proliferation [108]. In addition, METTL14 plays an important role in regulating chemoresistance, which seriously limited the efficacy of chemotherapy, the main clinical treatment for gastrointestinal cancer. In PC, METTL14 knockdown enhanced sensibility of cancer cells to cisplatin, promoting apoptosis and autophagy by repressing AMPKα, ERK1/2 and mTOR signal pathways [101]. Similarly, inhibition of METTL14 enhanced the gemcitabine sensitivity of PC cells by downregulating CDA [122]. METTL14 also mediated chemoresistance of CRC cells to cetuximab and HCC cell to sorafenib [103]. Given the crucial role of METTL14 in gastrointestinal cancer, it is urgently expected to screen, design, and develop effective METTL14 inhibitors and activators. Additionally, exploring drugs targeting upstream or downstream molecules of METTL14 may be also an effective measure for gastrointestinal cancer therapy. The combined applications of METTL14 inhibitor or activators with chemotherapy or immunotherapy show great potential as a promising treatment strategy and are anticipated to be investigated in the future.

## Discussion

m6A methylation is the most abundant RNA modification and has become research hotspot in recent years. m6A methylation affects the processing of mRNA and non-coding RNA and is of great significance for gene expression regulation. Mounting evidence indicated that m6A modification plays a critical role in tumorigenesis and progression. The present review showed the expression, function, and the regulatory mechanism of the methyltransferase METTL14 in gastrointestinal cancer, suggesting that METTL14 might be a promising biomarker for clinical diagnosis and therapeutic target of gastrointestinal cancer. However, with breakthroughs made in various aspects, contradictions and uncertainties have also been exposed, which is mainly consist of the following situations. (1) In different cancers, METTL14 has a dual regulatory effect on tumors. It serves as an oncogene in PC, while plays a suppressive role in HCC, CRC, and GC. Such complexity highlights that attention should be given to the application of METTL14 activators or inhibitors in case of inducing other tumors. (2) In different cancers, the expression level of METTL14 varies a lot. Some upstream regulations have been identified in specific cellular context, at transcriptional, posttranscriptional, and posttranslational level. However, the underlying rationales of whether it is upregulated or downregulated remain elusive. (3) For the same cancer, different researchers hold the opposite conclusions of METTL14. For example, Fan et al. found that overexpressed METTL14 inhibited proliferation and metastasis of GC [118], but Hu and his colleagues proved that METTL14-mediated upregulation of Linc01320 promotes GC cells proliferation, metastasis [121]. (4) For the same cancer, different studies showed the inconsistent results of METTL14. For instance, Dong et al. showed that inhibition of METTL14 resulted in CD8^+^ T cells dysfunction and promoted malignant progression of CRC [109]. Nevertheless, Wang et al. indicated that METTL14 deletion enhanced the efficiency of CD8 + T cells and elevated the immune response of CRC [108]. To sum up, multi-center large-scale studies are extremely required to further determine the role of METTL14 in gastrointestinal cancer, which could lay a foundation for precise individualized treatment.

Although great advances have been achieved in revealing the functions and regulatory mechanisms of METTL14, some problems need to be further explored. (1) As an integrity of m6A methyltransferase complex, METTL14 and METTL3 are supposed to have synergistic effects. However, many findings have demonstrated the opposite expression and functions between them in various cancers. Also, different targets and regulatory mechanisms of METTL3 and METTL14 have been proved. We hypothesized that they have biological functions independent of the methyltransferase complex, with their own bias towards targets. Therefore, the structural basis and regulatory roles of the METTL3/14 complex, and their respective functional mechanisms require further experimental verification. (2) As a promising biomarker for tumor clinical diagnosis, the sensitivity and specificity of METTL14 need to be further clarified. (3) Previous studies suggested that METTL14 may be a potential therapeutic target for gastrointestinal cancer, but insufficient attention was paid to drug development and no specific chemotherapeutic agent targeting METTL14 has been reported in both experimental researches and clinical practice so far. It is worth to determine the validity and feasibility of METTL14-targeted agents alone or in combination with existing therapies for treating tumors in the future. Importantly, special emphasis shall be given on the development of METTL14 inhibitors or activators in cancer treatment due to the double-edged sword roles of METT14 in gastrointestinal cancer.

## Conclusion

In summary, METTL14 plays an important role in gastrointestinal cancer and it may serve as a promising diagnostic/prognostic biomarker and a potential therapeutic target. We anticipate more future researches to further explore the therapeutic potential of METTL14 for feasible application in clinical practice.

## Data Availability

Not Applicable.

## Abbreviations

3’UTRs: 3’ Untranslated regions
ACLY: ATP citrate lyase
ALKBH3/5: AlkB homolog 3/5 RNA demethylase
Ankle1: Ankyrin repeat and LEM domain-containing protein 1
AKT1S1: AKT1 substrate 1
ARRDC4: Arrestin domain-containing protein 4
CLK1: Cdc2-like kinases 1
CRC: Colorectal cancer
CSAD: Cysteine sulfonate decarboxylase
CXCL19: Chemokine (C-X-C motif) ligand 19
DGCR8: DiGeorge syndrome critical region 8
EBI3: Epstein-Barr virus induced 3
EGFR: Epidermal growth factor receptor
eIF: Eukaryotic initiation factor
EMT: Epithelial-mesenchymal transition
ER: Endoplasmic reticulum
Flacc: Fl(2)d-associated complex component
FOXO3a: Forkhead box O3a
FTO: Fat mass and obesity-associated protein
GC: Gastric cancer
GEO: Gene Expression Omnibus
GOT2: Glutamic oxalacetic transaminas
HCC: Hepatocellular carcinoma
HNF-3γ: Hepatocyte nuclear factor-3γ
HNRNP: Heterokaryotic nuclear RNA protein
HRD1: HMG-CoA reductase degradation protein 1
HuR: Human antigen R
IFN-c: Interferon-c
IGF2BP: IGF2 mRNA binding protein
KDM5C: (K)-specific demethylase 5C
LC: Liver cancer
m1G: 1-Methylguanosine
m5C: 5-Methylcytosine
m6A: N6-methyladenosine
METTL3/14/16: Methyltransferase-like 3/14/16
NAFLD: Non-alcohol Fatty Liver Disease
OATP1B: Organic anion-transporting polypeptide 1B
PC: Pancreatic cancer
PIK3CB: Phosphatidylinositol-4,5-bisphosphate 3-kinase catalytic subunit beta
RBM15/15B: RNA-binding motif protein 15/15B
SCD1: Stearoyl-CoA desaturase 1
SIRT6: Sirtuin 6
SLC7A11: Solute carrier family 7 member 11
SOCS2: Suppressor of cytokine signaling 2
SOX4: SRY-box transcription factor 4
SPI1: Transcription factor PU.1
SRSF5: SR-like splicing factors 5
TCF4: Transcriptional factor 4
TCGA: The Cancer Genome Atlas
USP48: Ubiquitin specific peptidase 48
VIRMA/KIAA1429: Virlike m6A methyltransferase-associated
WTAP: WT1-associated protein
YTHD: YTH domain family of protein
ZC3H13: Zinc finger CCCH-type containing 13
